# Follow-up Analysis of Serum TNF-Related Apoptosis-Inducing Ligand Protein and mRNA Expression in Peripheral Blood Mononuclear Cells from Patients with Ischemic Stroke

**DOI:** 10.3389/fneur.2018.00102

**Published:** 2018-03-05

**Authors:** Kemal Ugur Tufekci, Ufuk Vurgun, Onur Yigitaslan, Pembe Keskinoglu, Erdem Yaka, Kursad Kutluk, Sermin Genc

**Affiliations:** ^1^Izmir International Biomedicine and Genome Institute, Dokuz Eylul University, İzmir, Turkey; ^2^Department of Neuroscience, Institute of Health Sciences, Dokuz Eylul University, İzmir, Turkey; ^3^Department of Neurology, Faculty of Medicine, Dokuz Eylul University, İzmir, Turkey; ^4^Department of Biostatistics, Faculty of Medicine, Dokuz Eylul University, İzmir, Turkey

**Keywords:** stroke, TNF-related apoptosis-inducing ligand, biomarker, ischemia, follow-up

## Abstract

Tumor necrosis factor (TNF)-related apoptosis-inducing ligand (TRAIL), which is TNF receptor superfamily member, contributes to several diseases pathogenesis. The aim of this research was to investigate the relevance of serum TRAIL protein levels and mRNA expression in peripheral blood mononuclear cells (PBMC) of patients with stroke through 6 months follow-up. We enrolled patients with first-ever acute ischemic stroke (*n* = 95) and healthy controls (*n* = 95) in this study. Follow-up blood samples were collected from patients at day 7, 28, and 180 after the onset. The stroke severity was evaluated by National Institutes of Health Stroke Scale score. TRAIL protein levels were quantified by using ELISA kits and TRAIL mRNA expression by quantitative real-time PCR. Our study showed that stroke patients have statistically significant lower levels of serum TRAIL protein (*p* < 0.0001) and elevated TRAIL mRNA expression (*p* < 0.0001) in PBMC at the disease onset. Our follow-up study revealed that TRAIL protein levels were increased while mRNA expression levels were downregulated in later periods. Overall, our findings suggest that serum TRAIL levels and mRNA expression in PBMC could reliably serve as a predictor of stroke outcome. Additionally, our study supports that TRAIL plays a role in pathogenesis and progression of ischemic stroke.

## Introduction

Tumor necrosis factor (TNF)-related apoptosis-inducing ligand (TRAIL) is a cytokine expressed by a variety of immune cells, and it was found to be upregulated upon stimulation with particular antigens (1). Previous reports have shown that TRAIL can bind to five specific receptors, two of which, particularly DR4 and KILLER/DR5, possess a conserved death domain that trigger apoptosis and inflammatory responses (1, 2). However, TRAIL shows protective consequences by inducing proliferation of vascular endothelial cells. Proliferative effects are mediated by three other receptors acting as decoy proteins, namely, TRAIL-R3/DcR1/TRID, TRAIL-R4/DcR2, and osteoprotegerin (3, 4). This summarizes that TRAIL has distinct functions and varies depending on the cell type and signaling pathway (5).

Many previous clinical studies have pointed out that reduced serum TRAIL levels show an association with unfavorable prognoses in different cardiovascular diseases (6–10). Thus far, there is a limited number of reports which precisely describe the relationship based on elevated/reduced serum TRAIL levels in cerebral ischemic patients. Pan and Colleagues show that serum TRAIL levels were reported to be lower in patients with large-artery atherosclerotic stroke in comparison to healthy individuals (11). Furthermore, Kang and Colleagues additionally show that the level of serum TRAIL is negatively correlated with National Institutes of Health Stroke Scale (NIHSS) score and stroke volume (12). However, there are no available reports which identify the variation in TRAIL mRNA expression pattern during and/or post onset in patients associated with ischemic stroke. In our present study, we investigated the levels of serum TRAIL protein and mRNA in peripheral blood mononuclear cells (PBMC) both in acute ischemic stroke patients and healthy controls. In addition, we also performed serum TRAIL and mRNA expression analyses during a time course follow-up period in stroke patients.

## Materials and Methods

### Subjects

The study received institutional ethics approval from Dokuz Eylul University School of Medicine (protocol no: 72-SBKAEK). Structured written consent was obtained from all participants by researchers. Patients with first-ever acute ischemic stroke admitted to Dokuz Eylul University Hospital in Izmir within 24 h after stroke onset were included in this study. Cerebral infarction was confirmed by computerized tomography or magnetic resonance imaging of the brain in all the enrolled patients. The subtypes of acute ischemic stroke patients were determined by Trial of Org 10172 in Acute Ischemic Stroke Treatment (TOAST) criteria as cardioembolism, large-artery atherosclerosis (LAA), small-vessel disease, and other determined/undetermined etiology (13). The severity of patients was determined by NIHSS (14). Healthy volunteers from the same geographical area who had no previous ischemic or other neurological disorders were enrolled in this study. All cases and controls were matched into gender and 10-year age groups.

### Clinical Data

Demographical data such as age, gender, height, weight, body mass index (BMI; kg/m^2^), and classical risk factors, such as hypertension, diabetes, hypercholesterolemia, smoking, alcohol consumption, were collected from both patients and healthy controls. Hypertension was diagnosed as systolic blood pressure ≥140 mmHg or diastolic blood pressure ≥90 mmHg on repeated measurements or current use of anti-hypertensive medication. Diabetes as a risk factor was recorded depending on patients’ self-report of a previous physician diagnosis or use of insulin/oral hypoglycemic agents. Current smoking was defined if the participant had smoked more than 100 cigarettes in their lifetime and also smoked in last 30 days upon participation in the study. Alcohol consumption was defined if the participant had consumed three or more standard drinks weekly (15). Use of antiaggregant/anticoagulant was determined when the participant used within 3 months due to non-stroke reasons.

### Blood Sampling, Purification, and Storage

Blood samples were collected from patients within 24 h after stroke onset followed by the 1st week (7th day), the 1st month (28th day), and the 6th month (180th day). Blood samples were also collected from age-matched healthy controls only at one time point. Blood sera were separated by centrifugation at 2,000 *g* for 10 min. PBMC were isolated by Biocoll (Biochrom, Germany) density gradient centrifugation. Sera and PBMC samples were stored at −80°C until analyses.

### Determination of Serum TRAIL Level

Serum TRAIL protein levels were determined by ELISA kit (R&D Systems, Minneapolis, MN, USA) according to manufacturer’s instructions. The absorbance values were measured at 450 nm using a microplate reader (Varioskan, Thermo Scientific).

### Determination of TRAIL mRNA Expression

Total RNA was isolated from PBMC of patients and control samples using miRNeasy Mini Kit (Qiagen, CA, USA) following the manufacturer’s instructions. RNA concentration and quality were determined by using a Nanodrop spectrophotometer (NanoDrop 2000, Thermo Scientific). First strand cDNA synthesis was carried out by reverse transcription of 500 ng of total RNA using miScript II RT Kit (Qiagen, Germany). TRAIL and β-actin mRNAs were amplified using LightCycler Primer Set (Search-LC, Germany) with the LightCycler FastStart DNA Master Plus SYBR Green I kit (Roche, Basel, Switzerland) according to manufacturer’s protocol. TRAIL mRNA expression was normalized to β-actin as housekeeping gene. The relative expression level of TRAIL mRNA was calculated by the 2^−ΔΔCt^ method (16).

### Statistical Analysis

Statistical analysis was performed using SPSS 23.0 for Windows. Data for continuous variables were presented as means ± SD and for categorical variables were presented as frequency. The difference between the serum TRAIL protein of patients and controls were analyzed using Student’s *t*-test. TRAIL mRNA expression differences between the two groups were compared using Mann–Whitney *U* test. The relation between the TRAIL levels and categorical variables were analyzed using Student’s *t*-test. The relation between the TRAIL levels and continuous variables were analyzed with Spearman’s correlation method. Repeated measurements of serum TRAIL and mRNA expression in PBMC were compared with Friedman variance analysis.

A receiver-operating characteristic (ROC) curve analysis was used for determination of the predictive power and estimation of optimal diagnostic cutoff value of serum TRAIL protein levels and TRAIL mRNA expression in PBMC. To test the hypothesis that the Area under the ROC curve (AUC) is 0.5, AUC and 95% confidence intervals were used to determine the diagnostic value of the TRAIL levels. Multivariate logistic regression analysis was performed to assess the independent association of comorbidities, lipid profiles, and TRAIL levels with stroke. Statistical significance value was accepted as *p* < 0.05.

## Results

We enrolled ischemic stroke patients (*n* = 95) and healthy controls (*n* = 95), who fulfilled inclusion criteria. Clinical characteristics of all participants are shown in Table 1. There were no differences between patients and controls in terms of mean age, gender, BMI, or alcohol consumption frequency. Consequently, the presence of hypertension, diabetes, smoking, and use of antiaggregant/anticoagulant was significantly higher in patients compared to controls (*p* < 0.05).

**Table 1:** Clinical characteristics of stroke patients and controls.

Variables	Controls (*n* = 95)	Stroke Patients (*n* = 95)	*p*-Value*
Gender, male *n* (%)	47 (39.4)	57 (58.6)	0.155
Age (mean ± SD)	69.65 ± 8.12	67.35 ± 10.13	0.085
BMI (mean ± SD)	27.16 ± 3.58	27.44 ± 3.25	0.582
Hypertension *n* (%)	42 (44.2)	70 (75.3)	**<0.001**
Diabetes *n* (%)	11 (11.6)	23 (24.7)	**0.012**
Smoking *n* (%)	31 (32.6)	47 (50.5)	**0.006**
Alcohol *n* (%)	21 (22.1)	19 (20.4)	0.922
Antiplatelet/anticoagulant *n* (%)	25 (26.3)	36 (38.7)	**0.038**
National Institutes of Health Stroke Scale Score (mean ± SD)		8.95 ± 5.64	
Total cholesterol (mean ± SD)	205.9 ± 39.58	197.7 ± 47.69	0.204
HDL cholesterol (mean ± SD)	50.1 ± 15.99	48.3 ± 16.75	0.472
LDL cholesterol (mean ± SD)	115.6 ± 31.34	110.2 ± 36.17	0.287
Triglyceride (mean ± SD)	144.4 ± 61.46	161.4 ± 90.30	0.138
Serum TNF-related apoptosis-inducing ligand (TRAIL) level (pg/ml) (mean ± SD)	156.7 ± 50.50	83.99 ± 23.43	**<0.0001**
TRAIL mRNA fold (mean ± SD)	1.638 ± 1.628	9.36 ± 10.64	**<0.0001**

*The p-value was calculated by comparing controls and stroke patients.

*The significant comparisons are displayed in bold*.

**Figure 1:**
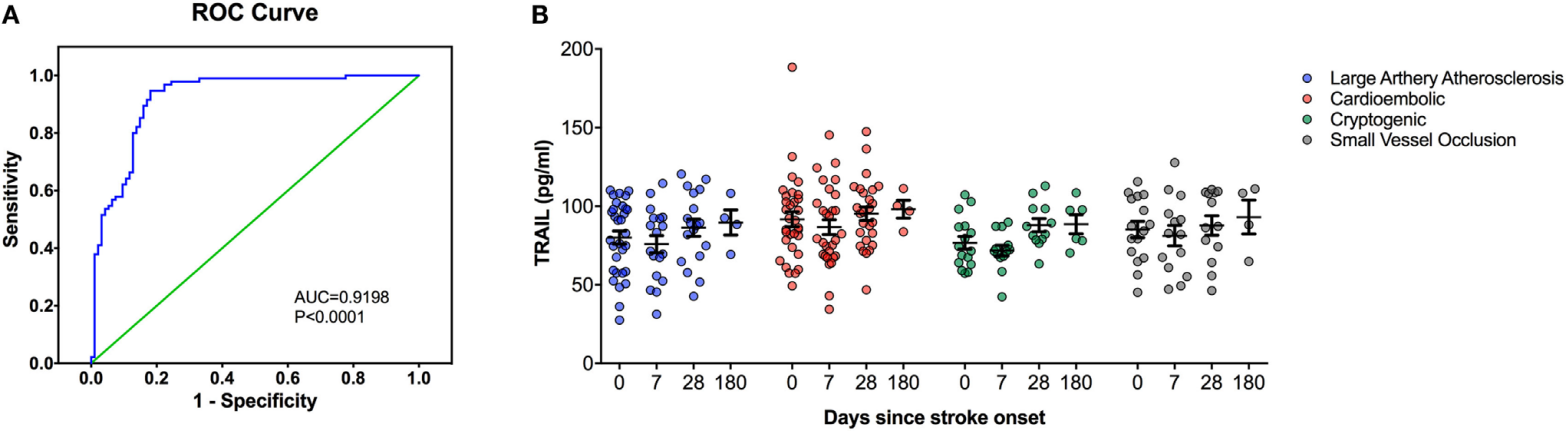
Serum TNF-related apoptosis-inducing ligand (TRAIL) protein level analysis in stroke patients. **(A)** Receiver-operating characteristic curve (ROC) analysis of predicted sensitivity and 1-specificity with serum protein levels of TRAIL. The area under the ROC curve (AUC) for TRAIL for stroke was 0.9198, and the optimal cutoff value for TRAIL was 107.32 pg/mL. **(B)** Serum TRAIL levels according to Stroke Subtype during the follow-up.

**Figure 2:**
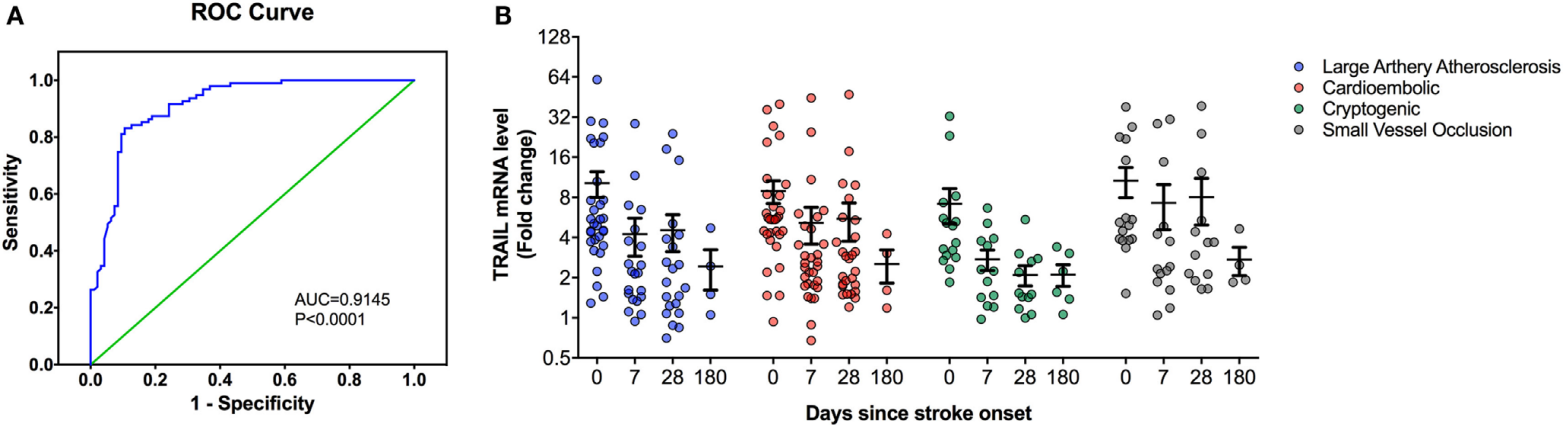
Peripheral blood mononuclear cells (PBMC) TNF-related apoptosis-inducing ligand (TRAIL) mRNA expression analysis in stroke patients. (**A**) Receiver-operating characteristic curve (ROC) analysis of predicted sensitivity and 1-specificity with PBMC mRNA levels of TRAIL. The area under the ROC curve (AUC) for TRAIL for stroke was 0.9145, and the optimal cutoff value for TRAIL was 3.16-fold. **(B)** PBMC TRAIL mRNA expression according to Stroke Subtype during the follow-up.

At the time of admission, serum TRAIL levels of stroke patients were statistically lower than healthy controls (<0.0001) (Table 1). When serum TRAIL protein levels in patients with different stroke subtypes were compared, no statistically significant difference was observed within first 24 h. Interestingly, follow-up analysis showed that serum TRAIL levels were significantly increased after 1 month of stroke onset (*p* = 0.002) (Figure 1B). Moreover, the stroke patients had significantly higher TRAIL mRNA expression in PBMC as compared to the controls (<0.0001) (Table 1). We did not find any significant differences in TRAIL mRNA levels between subtypes of stroke. In the follow-up analysis, TRAIL mRNA expression in PBMC were found to be decreased as compared to the first week of stroke onset (*p* < 0.001) (Figure 2B). We did not determine any significant correlation between TRAIL levels and stroke severity. Additionally, there were no correlations between serum TRAIL levels versus stroke risk/protective factors, such as hypertension, diabetes, smoking, alcohol consumption, and antiaggregant/anticoagulant use.

We next performed the ROC analyses to test whether TRAIL levels can discriminate stroke from the controls. Our data showed that, serum TRAIL levels had diagnostic value for stroke (AUC = 0.9198, 95%; 0.8793–0.9603, *p* < 0.0001) (Figure 1A). Sensitivity and specificity at the cutoff value of <53.5 pg/ml, 75.8 and 89.3%. TRAIL mRNA expression in PBMC also had diagnostic prediction for stroke (AUC = 0.9145, 95%; 0.8743–0.9456, *p* < 0.0001) with sensitivity of 83.2% and specify of 89.5% at the cutoff value of 3.16-fold (Figure 2A).

Logistic regression analysis was performed to analyze the relationship between risk factors and stroke. Age, BMI, hypertension, diabetes, alcohol consumption, smoking, lipid profiles, and TRAIL levels were used as variables for a logistic regression analysis. Our model contains hypertension, smoking, and TRAIL serum levels as variable and explains 73% of all cases. Our model revealed that TRAIL is a strong determinant of stroke (*p* < 0.0001). Moreover, hypertension and smoking are positively associated with stroke cases, which indicate intervention effectiveness (*p* = 0.026 for both factors). However, no significant regression was found after other factors in the model.

## Discussion

TNF-related apoptosis-inducing ligand is a member of the TNF-superfamily of cytokines leading to apoptotic cell death through engagement of death receptors *via* extrinsic apoptosis pathway (17). TRAIL is expressed by myeloid cells and specifically microglia in central nervous system (1). Inflammatory insults, such as lipopolysaccharide and interferon gamma, have been shown to cause upregulation of TRAIL (1). Increased TRAIL expression is also related to some neurodegenerative diseases, such as Alzheimer’s disease (18). However, there is still considerable ambiguity with regard to the exact role of TRAIL in stroke.

TNF-related apoptosis-inducing ligand plays an essential role in vascular endothelial and smooth muscle cell physiology (5) The studies in endothelial cells, vascular smooth muscle cells, and inflammatory cells revealed that TRAIL-induced apoptosis and may regulate interaction between endothelial and inflammatory cells (19–21). There is controversial data for the role of TRAIL on vascular inflammation. It has been described that TRAIL exerts both proinflammatory and anti-inflammatory effect on endothelial cells (2, 22).

Our results suggest that stroke patients have lower levels of serum TRAIL protein and elevated TRAIL mRNA expression in PBMC at the disease onset. However, during follow-up phase, serum TRAIL levels were downregulated while, TRAIL mRNA expression in PBMC were found to be increased. However, comparison between serum TRAIL protein levels and PBMC TRAIL mRNA expression among stroke subtypes did not result in any statistical significance.

The present study shows that serum levels of TRAIL during first 24 h were significantly lower in patients with ischemic stroke as compared to the controls. In corroboration with our results, a previous study also found lower serum TRAIL levels in patients with LAA stroke within 7 days after the stroke onset (11). One of the possible reason for the decrease in TRAIL levels in the acute phase of stroke might be due to the proteolytic cleavage of TRAIL (e.g., MMP2) (23).

To the best of our knowledge, this is the first report demonstrating the mRNA expression changes in PBMC of ischemic stroke patients. Nakajima et al. (24) reported increased TRAIL mRNA expression in PBMC samples from acute cardiac ischemia patients. Increased TRAIL mRNA levels in PBMC may contribute to apoptotic and inflammatory processes in cerebral ischemia (25). A previous study showed that DR5, which is a TRAIL receptor, were highly increased in the cerebral cortices of rats 24 h after hypoxic ischemia (26). Therefore, enhanced TRAIL–DR5 interaction may lead progression of ischemia *via* enhancing apoptosis and inflammation.

Furthermore, the serum TRAIL changes during long-term follow-up period are firstly reported by our study. During the 6-month follow-up, serum levels of TRAIL were gradually increased. Previous studies showed a weak association between low plasma/or serum TRAIL level and stroke severity (11, 12). Although, we could not find any correlation between lower TRAIL concentration versus stroke severity.

We next sought to determine the association between serum TRAIL protein levels with the risk factors. However, there was no correlation between TRAIL levels versus stroke risk factors. Moreover, both serum TRAIL levels and mRNA expression in PBMC failed to distinguish subtypes of stroke.

Patient recruitment from a single center, small sample size, and lack of volumetric analysis of infarct area of the participants are the important limitations of our study. Additional studies need to be carried out in a larger patient cohort to validate the diagnostic potential of TRAIL.

In conclusion, our results provide evidence that the levels of TRAIL in serum were decreased and mRNA expression in PBMC were increased in patients with acute ischemic stroke and importantly these changes reversed during the follow-up periods. Our results support that serum TRAIL levels and/or TRAIL mRNA expression in PBMC hold potential for diagnostic biomarker in patients associated with acute ischemic stroke.

## Author Contributions

KT, UV, and SG conceived this study. OY, EY, and KK recruited patient samples and collected clinical data. KT and UV were primarily responsible for the biochemical and gene expression analyses. PK provided statistical analyses of the patient data and laboratory analyses. KT, UV, OY, PK, and SG performed analysis of the data. KT, UV, and SG prepared all tables and figures. KT, UV, OY, and SG performed the literature search. KT, UV, and SG wrote the manuscript and all other authors commented and approved the final manuscript. Final manuscript preparation and edits were performed by KT, PK, and SG.

## Conflict of Interest Statement

The authors declare that the research was conducted in the absence of any commercial or financial relationships that could be construed as a potential conflict of interest.
